# Young children display an increase in prosocial donating in response to an upwards shift in generosity by a same-aged peer

**DOI:** 10.1038/s41598-017-02858-y

**Published:** 2017-06-01

**Authors:** Emily J. E. Messer, Vanessa Burgess, Michael Sinclair, Sarah Grant, Danielle Spencer, Nicola McGuigan

**Affiliations:** Department of Psychology, School of Social Sciences, Heriot Watt University, Edinburgh, EH14 4AS UK

## Abstract

Adult humans frequently engage in the reciprocal exchange of resources with other individuals. However, despite the important role that reciprocity plays in maintaining co-operative exchange we know relatively little of when, and how, reciprocity develops. We first asked whether pairs of young children (*M* = 74 months) would engage in direct reciprocity in a ‘prosocial choice test’ where a donor could select either a higher, or a lower, value reward (1v 2) for a partner at no cost to themselves (1v 1). In a subsequent retest we asked, for the first time, whether young children increase their level of prosocial donating in response to an upwards shift in generosity from an initially selfish partner. In order to determine whether interacting with another child was fundamental to the development of reciprocity we included a novel yoked non-agent condition. The results suggest that the children were engaging in a calculated form of reciprocity where the prior behavior of their child partner influenced their subsequent level of donation days after the initial exchange. Crucially we show that the children were not influenced by the value of the rewards received *per se*, rather selection by a human agent was key to reciprocity.

## Introduction

Our everyday experience provides us with ample evidence that humans from many different cultural backgrounds show concern for other, even genetically unrelated, individuals^1^. A suite of studies have explored key questions regarding the ontogeny and phylogeny of such prosociality, including when and how prosocial tendencies develop, and whether the capacity for other regard is uniquely human, or shared with other species^2–6^. One facet of prosociality that has received a great deal of recent attention is resource donation, with researchers asking whether, and how, individuals distribute resources between themselves and other individuals^7–9^. Such distribution problems are of great importance as conflict over resources is likely to occur frequently in the lives of humans, and other species alike, with the choice to donate, or withhold, resources potentially impacting on an individual’s resource pool either directly through a lack of return donation in a service economy^10^, or indirectly through a loss of standing in the community^10, 11^. The current study aimed to provide a detailed exploration of the role that social influences play in the donating behavior of young humans. More specifically, we asked whether the level of prosocial donating in 3- to 8-year-old children is uniquely influenced by the donating behavior of their peers, before going on to ask, for the first time, whether children will increase their level of prosocial donating towards a peer who donated negatively in an initial exchange, if that same individual acted more generously in a subsequent exchange days later.

Recent studies have shown that both human^12, 13^, and non-human primates^14, 15^ are sensitive to inequalities in resource allocation, and frequently (but not always, see refs 16–18) match the value of the reward(s) allocated to them by a partner during reciprocal resource exchange^19, 20^. Previous research within the developmental literature has shown that whether or not children engage in reciprocity varies according to donor age, with 5-year-old children reciprocating in contexts that require the distribution of resources via co-operative turn taking^21^, as well as in contexts where donors are required to select the value of the resource allocated to their partner (fixed^17, 19^, or non fixed^20, 22^ payoffs). In contrast, younger preschoolers have shown less evidence of reciprocity, with 2-year-olds often allocating resources randomly^17^, and 3-year-olds displaying reciprocity in some studies^20^, but not others^22^. However, despite the studies described above making great inroads in detailing whether or not reciprocity occurs, the precise mechanisms underpinning any reciprocity displayed, and the way in which reciprocity develops beyond the age of 5 years, are yet to be fully examined. Indeed, with the exception of one study^17^ where no reciprocity was evident, previous examples of reciprocity have been restricted to contexts in which the participants were allowed to respond immediately, in an iterated manner, to their partner’s donating behavior, leaving open the question of the complexity of the mechanisms underlying such donating decisions, a gap that the current study aims to address.

Identifying the mechanisms that underlie such prosocial choices is of great theoretical interest, with potential explanations ranging from simple non-contingent mechanisms (symmetry based reciprocity, or attitudinal reciprocity), to more complex contingent mechanisms in which tit-for-tat score keeping of the prior behavior of other individuals occurs (calculated reciprocity)^11, 23–26^. In order to examine the mechanisms underlying reciprocity in early childhood we first asked whether pairs of children (3 to 8 years) would engage in tit-for-tat donating when given the opportunity to respond to their partner’s behavior. We built on performance in this initial exchange by asking, for the first time, whether low levels of prosocial donating in an initial exchange between two children would carry over to a second exchange (presented two days later), or whether initially low levels of prosociality would be overcome if the second exchange began with one child displaying an upwards shift in generosity. The inclusion of a large delay between each exchange moved beyond the immediate trial by trial exchanges employed in previous reciprocity studies, thus allowing the opportunity to more clearly detail the role of memory in the participants’ donating behaviour. In addition, by introducing a delay between the exchanges of two children from the same school we create a highly naturalistic context for reciprocity to develop, more directly simulating multiple interactions between children in their everyday environment. Importantly, alongside such child-to-child exchanges we included a novel yoked control condition, missing from all previous child studies, in which children received donations ‘selected’ by a non-human agent. The inclusion of this agent versus non-agent comparison is crucial in determining whether being the recipient of a donation made by human agent has a unique influence on children’s subsequent level of resource donation to a human partner. Without such a control we cannot convincingly show that any changes in prosociality are not simply attributable to receiving resources in general, but instead to specifically receiving resources from another human agent.

The resource distribution task used in the current study, the ‘prosocial choice test’ (PCT), was adapted from a task initially designed for use with chimpanzees^27^, and subsequently extended to other primate species^9^ and young humans^28–30^. The task, based on the principles of fixed choice economic decision making games, requires a donor individual to allocate rewards to themselves and a receiver by selecting between two alternative payoffs, where the choice of one payoff results in a more favourable reward allocation (higher value reward) for the receiver than the other (lower value reward). The task is typically presented using some, or all, of three different payoff structures where the donor can either opt for an egalitarian option where both individuals receive an equal value reward (1,1), or instead opt for a resource distribution that favours either: 1) the donor (prosocial variant; 1,1 vs 1,0), 2) the receiver (envy variant; 1,1 vs 1,2), or 3) requires the donor to sacrifice resources in order to make the more generous egalitarian choice (2,0 vs 1,1). Studies that have employed the resource distribution paradigm with children have shown that the level of prosocial donation varies according to the specific reward distribution used, with the greatest levels of generosity occurring in the prosocial variant and relatively low levels of prosocial donation occurring in the envy and costly variants^8, 30–32^. Performance in such tasks has also been shown to vary according to donor age with older children (aged 7–8 years), given no opportunity for reciprocation, making a greater number of prosocial donations than their younger counterparts^7, 8, 30^.

It could however be argued that the level of prosocial donation may have differed in these studies if the children had been allowed to engage in a series of reciprocal exchanges, rather than one individual acting exclusively as the donor without the possibility of retribution. Reciprocity in young children has been explored in a variety of contexts^33–37^, and in reciprocal versions of the PCT, where children alternate in the role of donor on a trial-by-trial basis, children as young as 5 years of age have shown themselves to be acutely aware of the value of the reward allocated to them by their partner, responding to a lack of generosity with a subsequent lack of generosity and vice versa^19, 38^. However, what remains unknown is the impact that these initial exchanges, particularly negative resource exchanges, have on future resource exchange between the same two individuals. Is it the case that pairs of children who begin by donating negatively to each other continue to respond negatively in future exchanges, or could a shift towards more prosocial donating by one partner lead to a subsequent increase in generosity within the pair?

In order to explore the impact that an upwards prosocial shift by one partner has on subsequent levels of generosity behaviour we built on the ‘prosocial test > social experience > prosocial retest’ paradigm used in a recent multi species (4- to 9-year-olds, and adult humans, capuchins, and chimpanzees) resource distribution study^7^. In an initial prosocial test phase Claidière *et al*.^7^ allowed one individual (A) the opportunity to allocate resources to a second individual (B), with no opportunity for B to reciprocate. Then, during a subsequent social experience phase an attempt was made to raise A’s level of generosity indirectly by placing those A donors who were initially less than generous to Bin the receiver role and exposing them to exclusively prosocial donations from two pre-trained peers (different to the original partner B), before A acted as donor for their original partner (B) once more (again with no opportunity for B to reciprocate). The results showed that the older children, and the chimpanzees, significantly increased their level of prosocial donations to B at retest (in prosocial payoff trials), suggesting that the high levels of prosociality shown by the pre-trained peers could indirectly increase the level of prosocial donating by the test participants(A) towards individual B.

We aimed to modify Claidière *et al*.’s^7^ social experience paradigm in two ways, first by directly training A to donate generously to B in the prosocial retest, and second by providing the opportunity for B to reciprocate at both test and retest. These modifications allowed us to detail whether an upwards shift in prosocial donating by one individual (A) can result in a corresponding increase in the level of prosocial donating by their partner (B) across repeated exchanges (presented a few days apart); a context that is arguably more naturalistic than one in which each individual acts exclusively as either the donor or the receiver. In addition allowing both individual A and B to act as both the donor and the receiver provided the opportunity to examine whether children recall, and act upon, changes in their partner’s behavior.

Furthermore the connection made by Claidière *et al*.^7^ between receiving prosocial donations and subsequently extending this prosocial experience to other individuals is somewhat premature as non-social influences could also explain the pattern of donating witnessed. It is for example possible that the increase in prosocial responding was not driven by the donor’s generosity *per se*, rather non-social factors, including an increase in the salience of, or the reinforcement obtained from receiving, the higher value reward^26^ may have led to the increase in prosocial donations witnessed in the retest phase. Therefore a key aim of the current study was to determine whether it is important that a human partner makes the prosocial selection, or whether it is merely receiving the higher value rewards, that influences the level of prosocial donation witnessed in such exchanges. This was achieved by including a non-social comparison, analogous to the ‘ghost’ demonstrations used in social learning studies, where an apparatus (e.g., a puzzle box) is ‘operated’ without human agency (e.g., controlled remotely by the experimenter using hidden wires), giving the impression to a naïve observer that the apparatus is moving by itself ^39, 40^ (Hopper, 2010^41^ for a review). We adapted the ‘ghost’ methodology in a non-agent condition, yoked to performance in the standard child-child (agent) condition, in which the ‘selection’ of the higher or the lower value reward was controlled remotely by the experimenter (i.e., a ghost donor). In doing so we provide, what is to our knowledge, the first rigorous examination of the role that human agency plays in influencing the levels of prosocial responding within dyads of human children.

**Figure 1:**
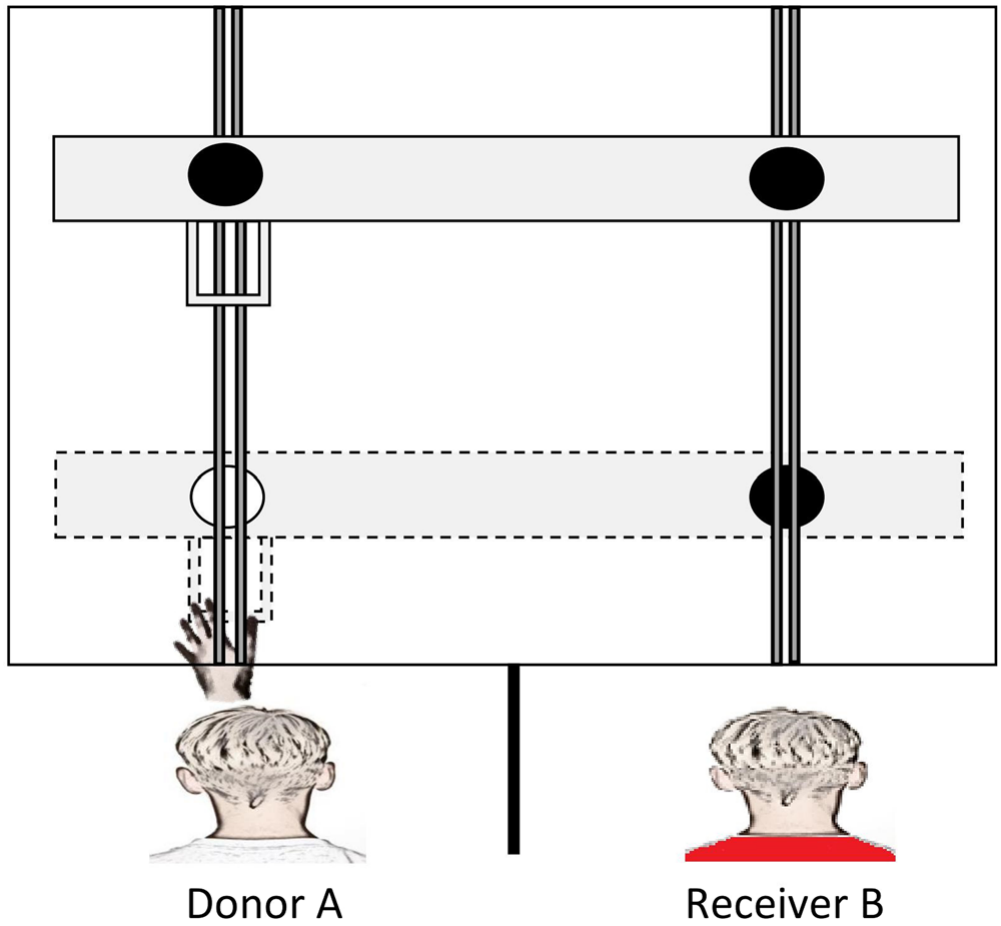
Bird’s-eye view of the experimental set-up used in the experimental trials of the agent condition. Note. Child A (the donor) is illustrated pulling the lower shelf forward using the handle attached to the box on the donor’s side of the apparatus. Child B (the receiver) is positioned in the receiver compartment awaiting the rewards coming into reach. The black circles represent the lower value rewards and the white circle the higher value reward.

In sum, the current study presented children with a PCT in which the rewards (stickers) were donated by either a human (see Fig. 1) or a non-human agent (see SI Fig. 1). In the test phase of the agent condition child A acted as a donor for child B, before the roles were reversed and B acted as donor for A. In the subsequent retest phase, presented two days later to require mental score keeping, we explored the influence of raising the levels of prosocial donating by pre-training child A to select exclusively prosocial choices for B, before the roles were reversed a second time. The yoked non-agent condition was equivalent to the agent condition with the exception that the donations made to child B at both test and retest were made by a non-agent (ghost A) donor rather than a human agent, while child A sat passively in the donor position. We predicted that the performance of child A and child B in the test phase of the agent condition would show evidence of reciprocal donating, i.e., a lack of generosity by A would be followed by a lack of generosity by B. In the retest phase of the agent condition we predicted that child B would increase their level of prosocial donating towards child A as a consequence of A’s increased generosity. In contrast, in the yoked non-agent condition we predicted that child B would be uninfluenced by the selection made by the donor (ghost A) at both test and retest. In addition to the experimental trials each participant completed (individually) two types of control trial, a self-centered control in which we tested for task understanding by allowing the participants to access the rewards on both the donor and receiver’s side of the apparatus, and an empty control where we determined how frequently each donor chose the higher value reward when no receiver was present (see SI Fig. 2). The envy distribution (1,1 v 1,2) was used exclusively throughout in order to ensure that the initial levels of prosocial donation by A were low enough to allow scope to increase A’s level of generosity in the retest phase.

## Results

### Overview of analysis

Pairs of same aged children (*n* = 48) were tested in either same gender, or opposite gender, pairs using the PCT employed in ref. 7. In an agent condition, each pair (*n* = 24 pairs) completed 16 prosocial test trials (8 trials with child A as donor, immediately followed by 8 trials with child B as donor), proceeded (two days later) by 16 prosocial retest trials (8 trials with child A pre-trained to act prosocially, immediately followed by 8 trials with child B as donor). The yoked non-agent condition (*n* = 24 pairs) comprised an identical structure to the agent condition with the exception that the rewards were delivered mechanically to child B while child A sat passively in the donor position. In addition to the prosocial trials both A and B completed (individually) a block of 8 empty control trials and a block of 8 self-centered control trials at both test and retest, totalling 48 trials for each participant (24 at test, and 24 at retest).

Of key interest in the analysis was: (1) whether the frequency with which B selected the higher value reward at test was contingent upon A’s donating behavior at test (2) whether the increased level of generosity displayed by A at retest would lead to an increase in the level of prosocial donating by B at retest (3) whether any contingent reciprocity witnessed was specific to the agent condition, and (4) whether B’s age influenced their donating behavior. In order to determine whether child B was donating reciprocally we conducted a binary logistic regression analysis (binary choice: 1 = higher value reward, 0 = lower value reward) on the data from the four prosocial test conditions (Agent: test/retest; Non-agent: test/retest). In the analysis of B’s test performance each binomial regression included two predictors: (1) A’s performance at test, and (2) B’s age (in months). The analysis of B’s retest performance included three predictors: (1) A’s performance at test, (2) B’s own performance at test, and (3) B’s age (in months). Full details of all regression analyses are provided in the Supplementary Analysis (see Table 1 and SI Table 1). In addition to the regression analyses a series of planned McNemar tests were conducted to determine whether there were any differences in the overall level of prosocial donating shown by A and B at test and retest.

**Table 1:** Coefficients of the model predicting B’s level of prosocial donating at test and retest in the agent condition [95% BCa bootstrap confidence intervals based on 1000 samples].

	b	95% CI for Odds Ratio		
		Lower	Odds	Upper
**Test Phase**				
Included				
Constant	1.45 [−0.83, 4.06]			
Child A test	0.72* [0.14, 1.34]	1.15	2.06	3.69
B’s age (months)	−0.02 [−0.06, 0.01]	0.95	0.98	1.01
**Retest Phase**				
Included				
Constant	−3.30 [−6.12, −0.91]			
Child A test	−0.65* [−1.41, 0.42]	0.27	0.52	1.00
Child B test	0.32 [−0.38, 1.01]	1.02	1.37	1.09
Child B’s age	0.06** [0.02, 0.10]			

Note. Test R² = 0.05 (Cox & Snell), 0.06 (Nagelkerke). Model χ²(2) = 9.24, p = 0.010

Note. Retest R² = 0.09 (Cox & Snell), 0.13 (Nagelkerke). Model χ²(3) = 18.22, p < 0.001.

Note. *p < 0.05; **p < 0.01.

As well as determining whether or not B’s behavior was equivalent to/contingent upon the prosocial choices made by A we were also interested in whether a cluster of participant characteristics influenced the level of prosocial donating by both A and B. The key characteristics of interest were the gender composition of the dyad, whether or not A and B were friends, as well as donor age. In order to determine whether these characteristics influenced the level of prosocial donating witnessed we conducted supplementary binomial regressions on performance in the experimental trials of the agent and non-agent conditions at both test and retest with the above characteristics as predictors. Full details of the supplementary analyses are provided in the Supplementary Information (see SI Analyses; SI Figs 5 and 6; SI Tables 5–8).

With respect to the control data we were interested in whether there were differences across trial type (experimental, self-centered control, or empty control) in the frequency with which the higher value reward was selected. In order to determine whether there were differences between the experimental trials and the control trials, we conducted a binary logistic regression analysis (binary choice: 1 = higher value reward, 0 = lower value reward) on the data from the four prosocial test conditions (Agent: test/retest; Non-agent: test/retest). In the analysis of both the test and the retest phase each binomial regression included trial type as a predictor of performance (self-centered control (SC), empty control (EC), and experimental (E)). Full details of all regression analyses are provided in the Supplementary Analysis (see SI Tables 2, 3 and 4). Follow up planned McNemar tests were conducted in order to determine where the specific trial differences lay.

### Agent Condition

#### Test Phase

**Figure 2:**
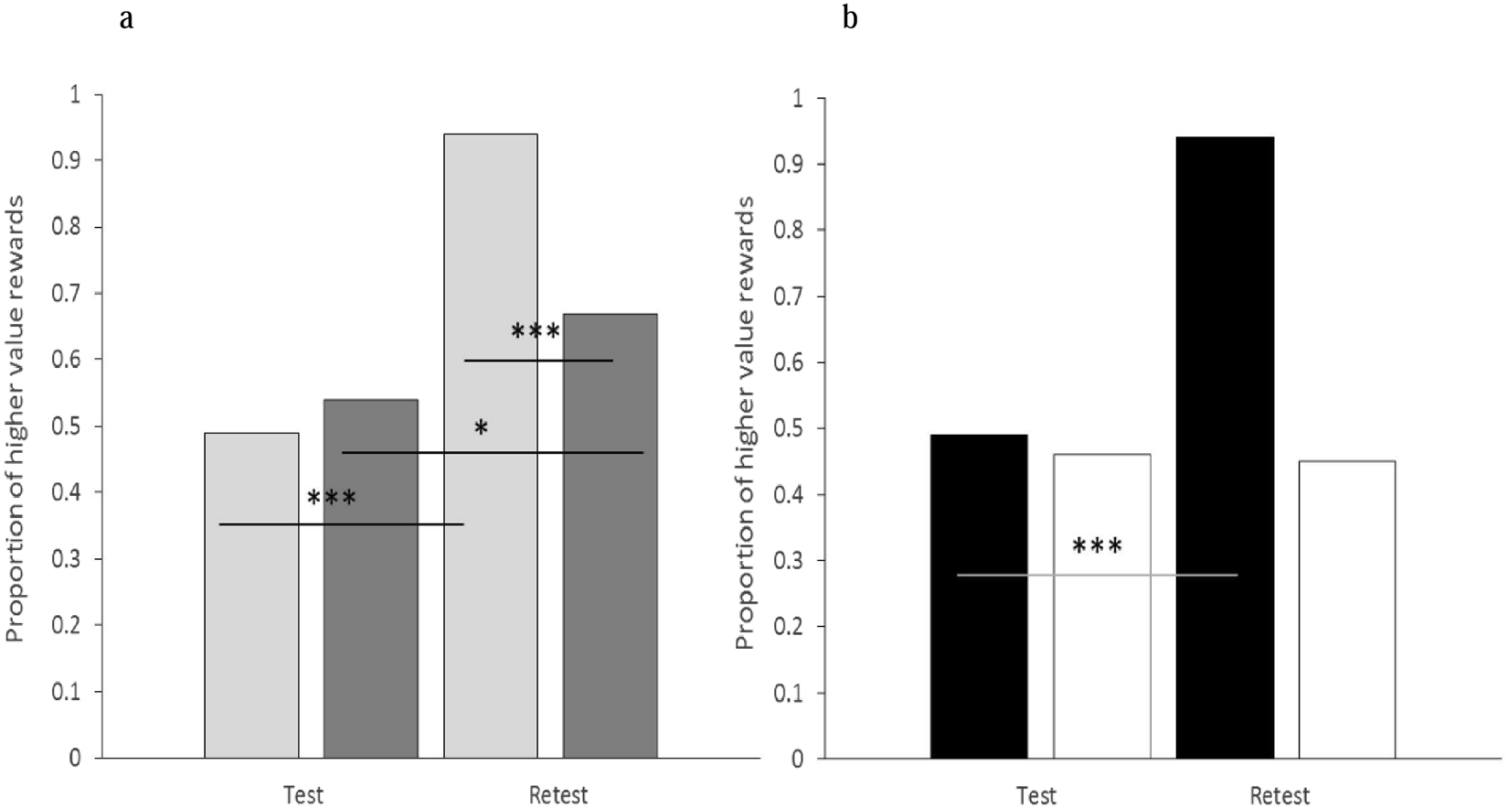
The proportion of higher value rewards selected by child B and child A/ghost A in the test and retest phases of the agent (panel a) and non-agent conditions (panel b). Note. In panel a the performance of child B is shown with dark gray bars and the performance of child A with light gray bars. In panel b the performance of child B is shown with white bars and the performance of ghost A by black bars. ***p < 0.001; *p < 0.05.

In the first exchange of the test phase, where child A acted as donor for child B, moderate levels of prosocial donating were witnessed, with children selecting an approximately equal proportion of lower and higher value rewards (child A to child B *M* = 0.49). When the roles were reversed, such that child B now acted as donor for child A, the number of prosocial choices made by B did not differ significantly from those selected by A (child B to child A *M* = 0.54; McNemar test, χ² = 0.63, p = 0.43; see Fig. 2). The similarity in the level of A and B’s prosociality appeared to be the result of reciprocal donating as the number of higher value rewards allocated by each individual was significantly and positively correlated (r = 0.19, p = 0.009). The contingent nature of B’s response was further highlighted by the significant logistic regression model (Wald χ² = 9.24, df = 2, p = 0.010; see Table 1), which revealed that the level of prosocial donating by A in the initial exchange significantly predicted B’s subsequent level of prosociality (p < 0.05), with B’s age having no influence on donating behavior.

#### Retest Phase

In the first exchange of the retest phase child A was pre-trained to allocate only higher value rewards to child B, with the result that the proportion of prosocial donations made by A increased significantly from test to retest (child A to child B test *M* = 0.49, child A to child B retest *M* = 0.94; McNemar test, χ² = 76.86, p < 0.001; see Fig. 2). When the positions were reversed, such that individual B was now in the donor role, the number of prosocial donations made by B increased significantly from that witnessed in the test phase (child B to child A test *M* = 0.54, child B to child A retest *M* = 0.67; McNemar test, χ² = 6.19, p = 0.013; see Fig. 2). However, despite the test-retest increase in the proportion of higher value rewards selected by B, the level of prosocial donating remained significantly lower than that displayed by A (McNemar test, χ² = 39.19, p < 0.001; see Fig. 2.), suggesting that although A’s increased level of prosociality led to an increase in B’s level of generosity it did not reach A’s retest level, a finding that is consistent with A’s earlier behavior influencing B’s level of generosity.

In order to explore the possibility that the somewhat disparate levels of prosociality displayed by child A and child B at retest was influenced by the level of prosocial donation in the test phase, we analysed B’s retest performance using a logistic regression that included both A’s level of prosocial donating at test, B’s own level of donating at test, as well as B’s age as predictors. The analysis revealed that the regression model was significant (Wald χ² = 18.22, df = 3, p < 0.001; see Table 1), with the level of prosocial donating by A (p < 0.05), but not B, in the test phase significantly predicting B’s level of prosociality at retest (p < 0.05), a finding which suggests that A’s initial level of donation constrained B’s choices in the second exchange. The analysis also revealed that B’s age predicted retest performance (p < 0.01), with children making increasingly generous donations with increasing age. Further support for the influence of A’s prior donating behavior on B’s retest performance comes from an analysis of only those A children (n = 8) who were spontaneously prosocial in the test phase (i.e., donated the higher value reward on 75% or more of trials). These generous A donors received an equivalent number of higher value rewards in return from their partner (number of higher value rewards A = 57, number of higher value rewards B = 46; χ²(1) = 1.18, df = 1, p = 0.28), suggesting that in an immediate context (with no prior exchange) B matched the high level of prosociality shown by A.

#### Prosocial Shift

Although the absolute number of higher value rewards delivered by child A at retest was not reciprocated by child B it is possible that the degree of change in A’s prosocial donating from test to retest influenced B’s level of generosity. In order to explore contingencies in A and B’s degree of change we calculated a ‘prosocial shift score’ for each participant that could range from −8 to +8, with positive values reflecting an increase in prosocial donating from test to retest, negative values reflecting a decrease in prosocial donating from test to retest, and 0 indicating no change between test and retest (See SI for more detail). Both child A (*M* shift = 3.6) and child B (*M* shift = 1.0) demonstrated a positive change from test to retest, a shift that was significantly and positively correlated (r = 0.25, p < 0.001; see SI Fig. 3). In order to further detail the link between A and B’s upwards shift in generosity we conducted a linear regression on B’s prosocial shift score including three predictor variables (A’s prosocial shift score, B’s age, and B’s recall of A’s test performance (exact match, close match (within 1 or 2 stickers), or no match (3 or more stickers out)). The analysis revealed that the model was significant (F = 9.80, df = 3, p < 0.001; see Table 2), with A’s prosocial shift score predicting B’s prosocial shift score (p < 0.05), and children becoming increasing likely to make a positive shift with increasing age (p < 0.001). The analysis also revealed that the extent to which B accurately recalled the number of higher value rewards donated by A at test marginally predicted the degree to which B displayed an upwards shift in prosociality (p = 0.079), suggesting a degree of calculation in the participant’s choices. Crucially, the accuracy of B’s recall correlated significantly with B’s prosocial shift score, even when B’s age was partialled out (r = −0.14, p = 0.046).

**Table 2:** Linear predictors of B’s prosocial shift from test to retest in the agent and non-agent conditions [95% BCa bootstrap confidence intervals based on 1000 samples].

	b	SE B	β	p
**Agent**				
Constant	−1.26 [−1.92, −.57]	0.35		
Child A’s prosocial shift	0.21* [0.02, 0.41]	0.10	0.16	p = 0.043
Child B’s memory score	−0.06 [−0.12, 0.01]	0.03	−0.14	p = 0.079
Child B’s age	0.02*** [0.01, 0.03]	0.01	0.32	p < 0.001
**Non-agent**				
Constant	−0.28 [−1.04, 0.49]	0.41		
Ghost A prosocial shift	0.07 [−0.14, 0.26]	0.10	0.06	p = 0.51
Child B’s memory score	0.7 [−0.03, 0.17]	0.05	0.14	p = 0.12
Child B’s age	0.002 [−0.01, 0.01]	0.01	0.03	p = 0.75

Note. Agent R² = 0.17, Model F(3) = 9.80, p < 0.001.

Note. Non-agent R² = 0.03, Model F(3) = 1.24, p = 0.30.

Note. *p < 0.05; ***p < 0.001.

### Yoked non-agent condition

#### Test phase

Of interest in the test phase of the non-agent condition was whether child B would match the value of the rewards delivered to them by a non-agent (ghost A) when subsequently acting as a donor for child A (who was initially passive in the donor compartment). The number, and sequence, of higher value rewards delivered by each non-agent was yoked exactly to those selected by a child donor (child A) in the agent condition (ghost A to child B *M* = 0.49). As in the agent condition the frequency with which the higher value reward was selected by B did not differ significantly from that selected by ghost A (child B to child A *M* = 0.46; McNemar test, χ² = 0.28, p = 0.60; see Fig. 2). However, in contrast to the reciprocity witnessed between child A and child B’s donating behavior in the agent condition the number of higher value rewards ‘donated’ by ghost A to child B, and subsequently by child B to child A, were not correlated (r = 0.06, p = 0.39), and ghost A’s test performance did not predict child B’s test performance (Wald χ² = 1.04, df = 2, p = 0.59; see SI Table 1), suggesting that child B was not donating to child A as a consequence of the value of the rewards allocated to them by ghost A. The analysis also revealed that B’s age had no influence on their donating behavior.

#### Retest phase

Of interest in the retest phase of the non-agent condition was whether the frequency of prosocial responding by child B would increase from test to retest when child B experienced elevated levels of generosity from a non-human agent (ghost A). As in the test phase the number of higher value rewards delivered by ghost A at retest were matched exactly to those selected by the pre-trained children (A) in the agent condition (ghost A *M* = 0.94). The results showed that in contrast to the pattern witnessed in the agent condition the increased number of higher value rewards ‘delivered’ by ghost A did not lead to a significant increase in B’s level of generosity towards child A (child B to child A test *M* = 0.46, child B to child A retest *M* = 0.45; McNemar test, χ² = 0.013, p = 0.91; see Fig. 2), suggesting that the non-agent partner was having little influence on B’s retest performance.

The logistic regression that included child B’s test and ghost A’s test performance (and B’s age) as predictors of B’s retest performance revealed that the full model was significant (Wald χ² = 9.55, df = 3, p = 0.02; see SI Table 1), with the performance of ghost A at test failing to significantly predict the level of prosocial donating by B at retest. However, B’s level of donating to child A at retest was predicted by B’s own level of donating in the test phase (p < 0.05), suggesting that B was responding consistently, and independently of, the ‘selections’ made by the ghost donor. The analysis also revealed that the level of generosity shown by B increased with increasing participant age (p < 0.05).

#### Prosocial shift

In contrast to the agent condition child B did not show a positive shift in their level of prosocial responding between test and retest (ghost A: *M* shift = 3.6, child B: *M* shift = −0.08) in response to the increased number of high value rewards provided by the non-human agent. This lack of shift was reflected in a non-significant correlation between ghost A and child B’s prosocial shift scores (r = 0.01, p = 0.85; see SI Fig. 3), with the linear regression analysis revealing a non-significant model (F = 1.23, df = 3, p = 0.20; see Table 2), with neither ghost A’s prosocial shift score, B’s age, or B’s accuracy in recalling the number of higher value rewards ‘allocated’ by ghost A significantly predicting B’s prosocial shift score.

## Control Trials

### Agent condition

#### Test phase

**Figure 3:**
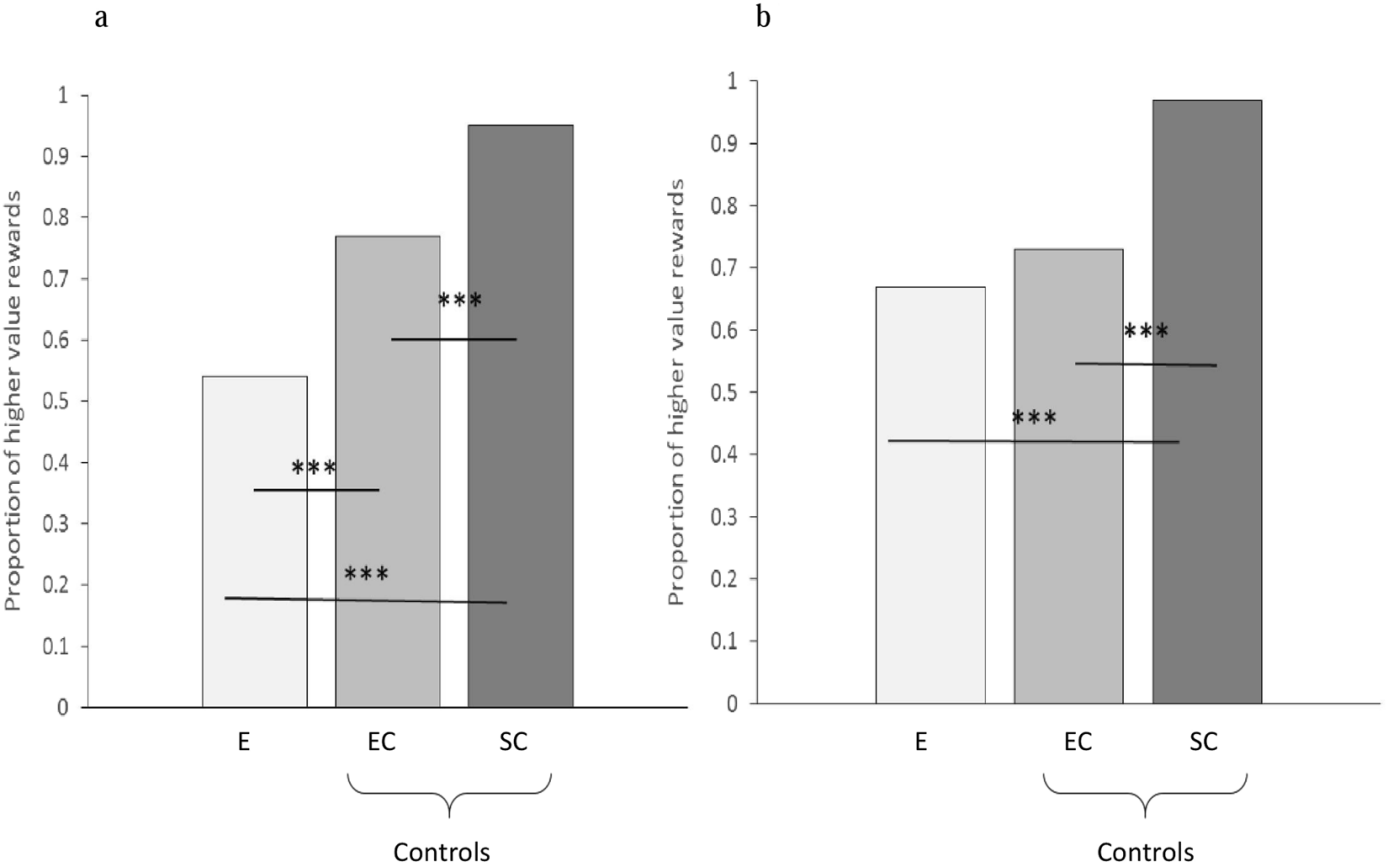
The proportion of higher value rewards selected by child B in the empty control, self-centered control, and experimental trials of the test (panel a) and retest (panel b) phases of the agent condition. Note. E = experimental trials, EC = empty control trials, SC = Self-centered control trials. ***p < 0.001.

Logistic regressions with trial type (self-centered control (SC), empty control (EC), and experimental (E)) as a predictor of A and B’s test behavior revealed that the frequency with which both child A (Wald χ² = 59.42, df = 1, p < 0.001; see SI Table 2), and child B (Wald χ² = 27.44, df = 1, p < 0.001; see SI Table 3), selected the higher value rewards varied significantly across trial type (child A: SC *M* = 0.94, EC *M* = 0.78, E *M* = 0.49; child B: SC *M* = 0.95, EC *M* = 0.77, E *M* = 0.54). Follow-up McNemar tests revealed that both A and B acted selfishly in the test phase, selecting the higher value rewards significantly more often in the empty trials (where no receiver was present) than in the experimental trials where a partner was present (child A: χ² = 32.76, p < 0.001; see SI Fig. 4; child B: χ² = 20.10, p < 0.001; see Fig. 3). In the self-centered control trials children displayed their task understanding, and motivation, by selecting the higher value rewards significantly more often than in either the empty trials (child A: χ² = 18.28, p < 0.001; see SI Fig. 4; child B: χ² = 25.69, p < 0.001; see Fig. 3), or the experimental trials (child A: χ² = 72.74, p < 0.001; see SI Fig. 4; child B: χ² = 66.86, p < 0.001; see Fig. 3).

#### Retest phase

Logistic regressions with trial type (SC, EC, and E) as a predictor of A and B’s retest behavior revealed that that the frequency with which child A (Wald χ² = 24.16, df = 1, p = <0.001; see SI Table 2), but not child B (Wald χ² = 2.25, df = 1, p = 0.13; see SI Table 3), selected the higher value rewards varied significantly across trial type (child A: SC *M* = 0.94, EC *M* = 0.77, E *M* = 0.94; child B: SC *M* = 0.97, EC *M* = 0.73, E *M* = 0.67). Follow-up McNemar tests revealed that the pre-training was successful, with A who had acted selfishly in the test phase, selecting the higher value rewards significantly more often for child B in the experimental trials than the empty trials in the retest phase (χ² = 19.22, p < 0.001; see SI Fig. 4). In contrast, the frequency with which B selected the higher value rewards in the experimental trials did not differ from that witnessed in the empty trials (p = 0.23), indicating that although B’s generosity had increased from test (where they acted selfishly) they were not acting prosocially at retest. Performance in the self-centered control trials revealed that the children remained extremely motivated, with the higher value rewards being selected significantly more often by both A and B in the self-centered control trials than in the empty control trials (child A: χ² = 20.89, p < 0.001; see SI Fig. 4; child B: χ² = 38.94, p < 0.001; see Fig. 3), and by B in the experimental trials (child B: χ² = 47.78, p < 0.001; see Fig. 3). The number of higher value rewards selected in the empty and self-centered control trials did not differ from test to retest suggesting that the motivation to obtain, or the salience of, the higher value reward remained consistent over time.

### Yoked non-agent condition

#### Test phase

**Figure 4:**
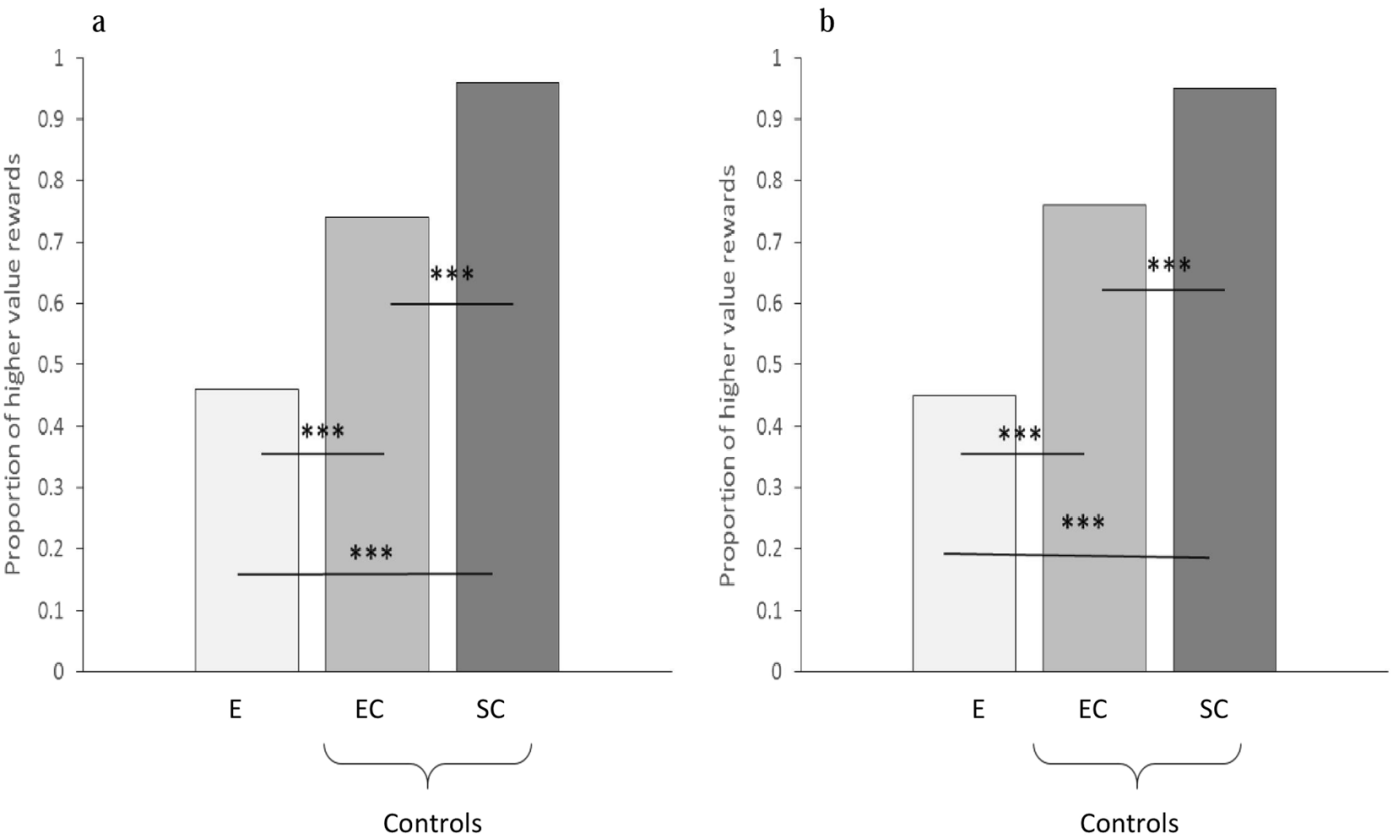
The proportion of higher value rewards selected by child B in the empty control, self-centered control, and experimental trials of the test (panel a) and retest (panel b) phases of the non-agent condition. Note. E = experimental trials, EC = empty control trials, SC = Self-centered control trials. ***p < 0.001.

As in the test phase of the agent condition the logistic regression analyses revealed that the frequency with which child B selected the higher value reward varied significantly across trial type (Wald χ² = 37.37, df = 1, p < 0.001; see SI Table 4). Follow-up McNemar tests revealed that the number of higher value rewards selected by B was significantly greater in the empty control trials (EC *M* = 0.74) than in the experimental trials (E *M* = 0.46, χ² = 27.89, p < 0.001; see Fig. 4), suggesting that as in the agent condition, child B was acting selfishly towards child A in the non-agent condition. The selection of the less generous option occurred despite the children demonstrating clear understanding of the task demands by selecting the higher value reward on the majority of the self-centered trials *(M* = 0.96), a tendency that was significantly greater than both the empty control trials (EC *M* = 0.74; χ² = 32.07, p < 0.001; see Fig. 4), and the experimental trials (χ² = 90.25, p < 0.001; see Fig. 4).

#### Retest phase

In contrast to B’s retest performance with a child partner, the logistic regression analyses revealed that there was a significant difference across trial type in the retest phase of the non-agent condition (Wald χ² = 46.61, df = 1, p < 0.001; see SI Table 4). Follow-up McNemar tests revealed that in contrast to the agent condition child B selected the higher value rewards significantly more often in the empty control trials (EC *M* = 0.76) than the experimental trials (E *M* = 0.45, χ² = 35.41, p < 0.001; see Fig. 4), suggesting that B continued to act selfishly towards A despite receiving an increased number of higher value rewards from the ghost donor. The number of higher value rewards selected in the self-centered control trials (SC *M* = 0.95) was again high, and significantly greater than that witnessed in both the empty control trials (χ² = 23.56, p < 0.001; see Fig. 4), and the experimental trials (χ² = 86.78, p < 0.001; see Fig. 4). Performance in the self-centered and the empty control trials did not differ between test and retest, suggesting that motivation, and attraction to the higher value reward remained consistent over time.

## Discussion

The current study shows, for the first time, that human agency plays a unique role in developing reciprocal exchange within dyads of young human children. In the test phase of the agent condition children (B), initially placed in the role of receiver, reciprocated the value of the rewards allocated to them by their child partner (A) when subsequently placed in the donor role. In a second set of exchanges (retest phase) the level of prosocial donating by the pre-trained A donors was elevated from that witnessed in the test phase, with the upwards shift in A’s prosocial donating leading to an increase in B’s generosity. However, despite B’s level of prosocial donating increasing at retest it did not reach the level of their pre-trained partner, with A’s earlier lack of generosity appearing to constrain B’s prosocial tendencies. Indeed, it appeared as though the degree of change in A’s behavior strongly influenced the tendency of B to act prosocially, whereby those donors (A) who showed the greatest change in prosociality from test to retest stimulated the greatest degree of positive change in B’s behavior. In contrast, despite the non-agent ‘donors’ (ghost A) allocating an identical number of rewards, in the same sequence, as the equivalent child (A) donors in the agent condition, the ‘selection’ by the non-agent had little influence on B’s subsequent donating behavior, suggesting that non-social factors were playing a very limited role in this context.

The reciprocity witnessed in the test phase of the agent condition would appear to be best explained by a *tit-for-tat* strategy, where the donating behavior of one individual was contingent on the level of donation made by their partner^19^. Alternative non-contingent mechanisms (i.e., symmetry based reciprocity, or attitudinal reciprocity) would appear to fit the pattern of performance less well. Attitudinal reciprocity occurs as the result of a short term mirroring of attitudes stemming from a perception that the individuals are jointly participating in a task, rather than responding to the specific choices made^24^. Key to the perception of behavioral co-ordination is that the time span between the responses of each individual is relatively short^26^. However, within the current study the responses of child B were delayed until child A had completed their block of trials, and yet despite the delay reciprocity prevailed, suggesting that a short term mirroring of attitudes was unlikely to explain the pattern of donating witnessed. Similarly, B’s performance in the retest phase was best predicted by the allocation provided by A in the initial exchange (that took place two days earlier), and to a lesser extent B’s ability to recall A’s earlier behavior, suggesting that the participants were most likely engaging in a calculated form of reciprocity, in which a mental record of the partner’s donating behavior from prior exchanges, was held over a relatively long time span.

An alternative non-contingent mechanism, symmetry based reciprocity, occurs in instances where the reciprocal exchange of resources is linked to mutual social preferences, with individuals preferentially directing aid to close associates^25^. Although all of the dyads in the current study were from the same school class, some dyads (friends) were more closely associated than others (merely acquaintances; see methods for more detail). If symmetry based reciprocity influenced the number of prosocial donations made then we would expect that pairs who were friends would display more generosity than pairs who were less closely associated. However, in the test phase of the agent condition those pairs who were friends were no more generous to each other than those pairs who were merely acquaintances, suggesting that reciprocity, at least in this initial exchange, was not based on close association. However, friendship did have an influence in the retest phase with children allocating significantly more higher value rewards to close associates than acquaintances, a finding that is consistent with recent studies that have shown that children prefer to donate to friends than non-friends^31, 32, 42^, and to members of their ingroup^30^. The selective increase in prosocial donating at retest suggests that the upwards shift in prosociality by friends was more likely to be rewarded than the equivalent increase by an acquaintance. It is possible that the ‘friend preference’ resulted from the children attempting to establish mutually beneficial co-operative exchanges with individuals who they were more likely to exchange resources with in the future. Alternatively, the children may have been seeking to be ‘fair’, or ‘egalitarian’, with their close associates, with children being more content for acquaintances, rather than friends, to receive a reduced share of the resource.

The relatively low levels of generosity displayed by the participants in the test phase are consistent with the findings of previous studies that have employed the envy distribution with children in this age range^7, 8, 30, 31^. Similarly, the significant increase in prosocial donating from test to retest both supports, and extends, the findings from the single study that has attempted to use positive social experience to enhance prosociality in a PCT, albeit in a less direct context^7^. In Claidière *et al*.^7^ those children who acted selfishly towards a partner in a test phase, were exposed to generous donations by two pre-trained children, before being retested with their original partner once more. The results showed that the social experience phase led to conditional increases in prosocial donating, with older children (7-year-olds), but not younger children (5-year-olds), increasing their level of prosocial donating in trials that employed the prosocial distribution (1,1 v 1,0), but not the envy distribution (1,1 v 1,2). The results of the current study support the main findings of Claidière *et al*.^7^ that social experience can enhance the level of prosocial donating in children, and that the influence of social experience was conditional on the age of the children. In the current study, the older children appeared to be more influenced by A’s shift in donating behavior than the younger children, perhaps as a result of an increased memory capacity allowing the older children to more accurately recall their partner’s earlier behavior. Crucially, by including a yoked non-agent control condition the current study goes beyond the previous data to demonstrate, for the first time, that it is social influences (i.e. a child actively donating resources to a partner), rather than non-social influences (i.e., receiving a preferred reward), that are crucial to elevating the levels of prosocial donating witnessed.

The findings of the current study also extend the influence of social experience to a different form of reciprocity from that explored previously. In Claidière *et al*.^7^, the test participants (A & B) remained exclusively in either the donor role or the receiver role, with different individuals (C & D) acting as the pre-trained models for B. The increase in prosociality in the retest phase is explained as a result of a tendency of the children to engage in generalized reciprocity- here defined as instances where individuals who experience prosocial acts directed to them by another individual are more likely to act prosocially towards other individuals^43–45^. This contrasts with the structure of the current study where the switching of roles between A and B allowed for the potential of direct reciprocity - where individual A acts prosocially towards B, because B was prosocial towards A previously^46^. Interestingly, the results of these two studies suggest that social experience may influence both direct and generalized reciprocity during the same developmental period, perhaps as a result of the children being at a stage where they are receptive to social experiences in this domain^7^.

However, it appears as though the specific reward pay-off employed has differential influences on the two forms of reciprocity. In the current study, where direct reciprocity was possible, children demonstrated an increased tendency to allocate the generous option at retest using the envy distribution, which contrasts with the generalized reciprocity paradigm where the prosocial distribution, but not the envy distribution, led to increased levels of generosity^7^. The differential pattern of performance with respect to the envy distribution may result from young children attempting to avoid being the recipient of disadvantageous inequity^13, 47^. In contexts that allow for direct reciprocity children already have prior experience of their partner’s generosity, and can adjust their return allocation accordingly, ensuring at least parity with that individual. This differs from a generalized reciprocity context where opting to allocate the generous option to another group member, may occur without information of that individual’s prosocial inclination, and could potentially result in the donor being the recipient of disadvantageous inequity that is never readdressed. In contrast, generalizing the generous selection in the prosocial pay-off (1,1) would appear less risky as this selection provides both partners with a higher value reward, rather than leaving the donor in the position of holding disadvantageous inequity. Future studies could usefully explore when, and under what pay-off conditions, the different forms of reciprocity emerge.

Overall, our findings suggest that young children, particularly those aged 6 years and upwards, are calculated donors who allocate rewards selectively depending on the prior donating behavior of their partner. In an initial test phase children responded to a lack of generosity with a subsequent lack of generosity, before increasing their level of prosocial donating in response to their partner exhibiting an upwards shift in prosociality at a later date. It appeared as though the decision to allocate the higher value reward stemmed from an ability to engage in mental score keeping, with children’s level of generosity being influenced by the allocation provided by their partner in an exchange that took place two days earlier. Thus the capacity to engage in calculated reciprocity, in which a record of a partner’s prior donating behavior is held over a long period, is likely key to the successful development of co-operative exchanges within groups of individuals, and thus marks an important developmental milestone. Future studies could usefully explore whether this move towards co-operation is reflective of ‘forgiveness’ on the part of the participants who were initially treated selfishly.

## Methods

### Apparatus

The apparatus used in the current study was identical to that presented to young children in a previous study^7^. The apparatus comprised a clear plastic box (L × W × H: 65 × 65 × 60 cm) that housed two plastic shelves (L × W: 57 × 7 cm), one positioned directly above the other. Handles were attached to the left hand side of each shelf (the donor’s side), whereas the right hand side (the receiver’s side) had no such handles attached. The area in front of the box was divided into a donor and a receiver compartment by attaching an opaque partition (H × W: 90 × 50 cm) at a perpendicular angle to the front of the box. From both the donor and receiver’s compartment, four rewards were visible; two on the donor’s side (one on the top shelf, and one on the bottom shelf), and two on the receiver’s side (one on the top shelf, and one on the bottom shelf). The rewards varied in value, either lower value (plain black sticker) or higher value (colorful sticker) and were of equal size. Each of the experimental and control trials used the envy pay-off structure (1,1 vs 1,2) in which the donor received the same lower value reward irrespective of which shelf they selected, whereas the donor always had a choice between allocating either a higher value reward, or a lower value reward, to the receiver. At the beginning of each trial both shelves were positioned outside of the participants’ reach, and required the donor to pull their chosen shelf towards both themselves and the receiver using one of the handles. The alternative shelf automatically retracted such that the rewards on that shelf were unobtainable once a selection had been made.

### Participants

The participants were 96 three- to eight-year-old children, 48 of whom were allocated to an ‘agent condition’ (27 females, 21 males; range = 46 to 95 months; *M* age = 74 months, SD = 11 months), with the remaining 48 children being allocated to a yoked ‘non-agent condition’ (28 females, 20 males; range = 49 to 99 months; *M* age = 74 months, SD = 11 months). An additional 48 children were tested but were not included in the final sample as they either failed to pass training (*N* = 37 children, 14 younger (<75 months,); 23 older (+75 months), were excluded due to an experimental error (*n* = 6), or failed to complete all phases of the experiment (*n* = 5). Participants were predominantly Caucasian and were recruited from, and tested in, primary (elementary) schools in Scotland, UK that served a variety of socioeconomic backgrounds.

Ethical approval was granted by the School of Life Sciences ethics committee at Heriot Watt University. Informed consent for all our participants was obtained via the child’s parent/legal guardian. All procedures were carried out in accordance with relevant guidelines and regulations.

### Procedure

#### Training Phase

The aim of the training phase was to ensure that the participants: (1) consistently viewed the colorful sticker as being of higher value than the plain sticker, and (2) that the participants understood, and consistently attended to, the relative value of the rewards on the receiver’s side of the apparatus. In a series of preference trials the participants could retrieve either a colorful sticker or a plain sticker from the donor’s side of the box. Those participants who selected the colorful sticker on 4 consecutive trials from a maximum of 10 trials were deemed to have a clear, and importantly pervasive, preference for the higher value reward and proceeded to the next stage of training (27 children failed the preference test, *M* number of trials higher value reward selected = 4.5). During the second stage of training the partition was removed and four rewards were placed inside the box, two on the donor’s side (two lower value rewards), and two on the receiver’s side (one higher value reward and one lower value reward). Those participants who were aware of, and attended to, the value of the rewards on the receiver’s side of the apparatus would be expected to repeatedly opt for the shelf that contained the greatest overall payoff (i.e., opt for 1,2 rather than 1,1). In order to proceed to the experimental phase the participants were required to demonstrate a clear, and pervasive, tendency to select the shelf containing the greatest overall payoff (4 consecutive trials from a maximum of 10). Ten children failed the second stage of training (*M* number of trials higher value reward selected = 4.2). We employed a relatively strict criteria for progression at both stages of training to ensure that the participants who entered the experimental phase were those who we could be confident were acting on the basis of reward value.

#### Experimental Phase

Participants who successfully completed training were allocated to either an agent condition, or a yoked non-agent condition, using a matched between participants design where the performance of A, and the age, and the gender (with the exception of one pair) of the pair were matched across the agent and non-agent conditions.

#### Test/Retest: Agent condition

In the agent condition pairs of same aged participants undertook a series of four exchanges in which they alternated in the role of donor and receiver for blocks of 8 experimental trials. In the first two exchanges of the test phase the choices made by both individual A and individual B were entirely spontaneous, and began with child A acting as a donor for child B (8 trials), before the roles were reversed so that child B acted as the donor in the second exchange (8 trials). The final two exchanges occurred in the retest phase, presented two days after the test phase, that followed the same structure as the test phase (i.e., A donated to B for 8 trials, then B donated to A for 8 trials). However, in contrast to the earlier exchange the choices made by A at retest were no longer spontaneous, as child A had, unbeknown to child B, been pre-trained to act prosocially.

#### Test/Retest: Non-agent condition

The choice of reward made (in the experimental trials at both test and retest) by each of the twenty-four A children in the agent condition was yoked to the value of the reward delivered by twenty-four ‘ghost’ A donors in a non-agent condition. In the non-agent variant the movement of the shelves occurred without the involvement of a human agent, rather the shelves were operated via a mechanical mechanism controlled remotely by the experimenter. Whilst the shelves were ‘moving of their own accord’ child A sat passively in the donor compartment (8 trials at both test and retest), before child B assumed the role as an active donor for A for 8 trials at both test and retest.

#### Control conditions

In addition to the experimental trials the participants completed a block of 8 empty control trials, and a block of 8 self-centered control trials in both the test and retest phases (order of control and experimental blocks counter-balanced). In the self-centered control the participant’s knowledge of the value of the rewards on the receiver’s side of the box was tested by allowing the participants to access the rewards from both compartments (partition removed). In an empty control we determined how frequently the participants chose the higher value reward when no receiver was present (partition in place), providing a direct comparison to the experimental trials where a receiver was present. Participants were deemed to have acted ‘selfishly’ if the frequency with which they selected the higher valued rewards was greater in the empty control than the experimental trials, and deemed ‘prosocial’ if the frequency with which they selected the higher valued rewards was greater in the experimental trials than the empty control trials. A block of each type of control was presented at both test and retest so that we could determine whether the children displayed any changes in motivation, or attraction to the higher value reward, with increasing exposure.

### Additional measures

#### Memory check

In order to determine whether the participants were keeping a mental note of their partner’s generosity child B was asked, upon completion of the retest phase, to recall how many higher value rewards child A/ghost A allocated to them at both test and retest.

#### Friendship

A measure of the relationship between the individuals in each pair was obtained by asking each class teacher to indicate whether the two individuals were friends or acquaintances.

## Electronic supplementary material

Supplementary Info
