# Supplementary material for: Young children display an increase in prosocial donating in response to an upwards shift in generosity by a same-aged peer
Source: Sci Rep. 2017 Jun 1;7:2633. doi: 10.1038/s41598-017-02858-y (PMC5453940; doi:10.1038/s41598-017-02858-y)
Supplement: Supplementary file 1 — Supplementary Info [file 41598_2017_2858_MOESM1_ESM.doc]

Young children display an increase in prosocial donating in response to an upwards shift in generosity by a same-aged peer

Emily J. E. Messer¹, Vanessa Burgess¹, Michael Sinclair¹, Sarah Grant¹, Danielle Spencer¹ and Nicola McGuigan¹*

¹Department of Psychology, School of Social Sciences, Heriot Watt University, Edinburgh, UK EH14 4AS

**Supplementary Information: Analyses**

Prosocial Shift Score

*Child A.* Child A was pre-trained to act prosocially towards B in the retest phase, resulting in a significant increase in prosocial donating by A from test to retest. Across the 24 pairs the vast majority (*n* = 20) of the A donors allocated the maximum number of 8 higher value rewards at retest, with another 3 donors allocating either 7 (*n* = 2), or 6 (*n* = 1) higher value rewards. The remaining donor allocated only 1 higher value reward at retest. An upwards shift in A’s prosocial donating could potentially range from a minimum of +1 (e.g., 7 higher value rewards donated at test, 8 higher value rewards donated at retest) to a maximum of +8 (i.e., 0 higher value rewards donated at test, 8 higher value rewards donated at retest). Those children who allocated an equal number of higher value rewards at both test and retest received a score of 0 (e.g., 8 higher value rewards donated at test, 8 higher value rewards donated at retest). Of the 24 A children 19 received a positive prosocial shift score (range = +1 to +8), and 4 received a score of 0. The remaining child showed a decrease in prosocial responding from test to retest (8 higher value rewards donated at test, 7 higher value rewards donated at retest) and received a prosocial shift score of -1.

*Child B.* The donating behavior of child B was entirely spontaneous at both test and retest, with positive prosocial shift scores having the potential to range from +1 (e.g., 5 higher value rewards donated to A at test, 6 higher value rewards donated at retest) to +8 (i.e., 0 higher value rewards donated at test, 8 higher value rewards donated at retest), and negative prosocial shift scores from -1 (e.g., 4 higher value rewards donated at test, 3 higher value rewards donated at retest) to -8 (i.e., 8 higher value rewards donated at test, 0 higher value rewards donated at retest). A prosocial shift score of 0 indicated that the child did not change their level of prosocial donation from test to retest (e.g., 2 higher value rewards donated at test, 2 higher value rewards donated at retest). Of the 24 B children 12 received a positive prosocial shift score (range = +1 to +8), 7 received a negative prosocial shift score (range = -1 to -7), and 5 received a score of 0.

### Influence of age, gender, and friendship on prosocial donating

The supplementary analyses aimed to determine whether donor age, friendship, and the gender composition of the dyad influenced the level of prosocial donating by child A and child B at test, and child B at retest. Child A’s performance at retest was not considered as the choices made by A were predetermined by the experimenter. In order to explore influences on B’s behavior we ran four binary logistic regressions (Agent test & retest; Non-agent test & retest) on the level of prosocial donating witnessed by B in the experimental trials of each phase (Binary choice: 1 = higher value reward; 0 = lower value reward). The four analyses each included three predictors: 1) the gender composition of the dyad (gender of donor indicated first): male A-male B (M-M), female A- female B (F-F), male A-female B (M-F), or female A-male B (F-M), 2) the age of child A or child B (in months), and 3) the relationship between the members of each dyad (friend (F), or acquaintance (A)). With respect to child A we conducted a single regression analysis (containing the 3 predictors outlined above) on A’s (spontaneous) behavior at test in the agent condition. Child A’s selections in the retest phase were not considered further as they were predetermined by the experimenter. Similarly, no analyses were conducted on the ‘choices’ made in the non-agent condition as child A was passive in this condition.

Agent Condition

*Child A*. The analysis of child A’s test performance shows that the full model was not significant (Wald χ² = 3.47, df = 3, p= .33; see SI Table 7), with the level of prosocial donating by A not varying according to the level of friendship between A and B (see SI Table 8), A’s age (see SI Fig. 5), or the gender composition of the dyad (see SI Table 8).

*Child B.* The analysis of child B’s test performance shows that the full model was not significant (Wald χ² = 4.08, df = 3, p = .25; see SI Table 9), with the level of prosocial donating by B not varying according to the level of friendship between A and B, B’s age, or the gender composition of the dyad. In the retest phase the analysis of B’s performance shows that the full model significantly predicted the level of prosocial donating shown by B (Wald χ² = 44.08, df = 3, p < .001, see SI Table 9), with friends receiving a significantly more generous allocation than acquaintances (A *M* = .52, F *M* = .71; p < .05; see SI Table 8), and older children (+ 75 months) allocating more generously than their younger (< 75 months) counterparts (younger B *M* = .55, older B *M* = .81, p < .01; see SI Fig 6), with additional McNemar tests showing that the level of prosociality shown by the older children increased significantly from test to retest (χ² = 17.8, p < .001; see SI Fig. 6). The model also revealed a significant influence of dyad gender (p < .001; see SI Table 8) with follow-up Mann Whitney U tests revealing that male-male dyads (*M* = .44) made significantly fewer prosocial choices than either female-female dyads (*M* = .67; p = .013), male-female dyads (*M* = .72; p = .014), or female-male dyads (*M* = .90; p < .001; see SI Table 8). In addition female-male dyads made significantly more prosocial choices than female-female dyads (p = .007) or male-female dyads (*M* = .72; p = .049; see SI Table 8). No other comparisons were significant.

Non-agent condition

*Child B*. The analysis of child B’s test performance in the non-agent condition shows that the full model was not significant (Wald χ² = 6.37, df = 3, p = .10; see SI Table 10), with the level of prosocial donating by B not varying according to the level of friendship between A and B, or the gender composition of the dyad. However, in contrast to performance in the agent condition, B’s age had a significant influence on test performance with children becoming increasingly prosocial with age (*M* younger = .44; *M* older = .49; p < .05; see SI Fig. 6). In the retest phase the analysis of B’s performance shows that the full model significantly predicted the level of prosociality shown by B (Wald χ² = 20.12, df = 3, p < .001, see SI Table 10), with B’s age influencing B’s behavior at retest (younger B *M* = .37, older B *M* = .55, p < .001; see SI Fig. 6). The analyses also showed that, in contrast with performance in the retest phase of the agent condition, the level of prosocial donating did not vary according to the level of friendship between A and B (see SI Table 8 ), or the gender composition of the dyad (see SI Table 8) in the non-agent retest.

**Supplementary Information: Figures**

Figure legends

SI Figure 1. Bird’s-eye view of the experimental set-up used in the experimental trials of the non-agent condition

SI Figure 2. Bird’s-eye view of the experimental set-up in the self-centered control trials (panel a), and the empty control trials (panel b)

SI Figure 3. Difference in the number of higher value rewards selected by B from test to retest as a function of the size of the difference in A’s test-retest allocation during the agent and non-agent conditions.

SI Figure 4. The proportion of higher value rewards selected by child A in the empty control, self-centered control, and experimental trials of the test (panel a) and retest (panel b) phases of the agent condition.

SI Figure 5. The proportion of higher value rewards selected by younger A (< 75 months) and older A (+ 75 months) in the experimental trials of the test phase of the agent condition.

SI Figure 6. Proportion of higher value rewards selected by younger B (< 75 months) and older B (+ 75 months) B in the experimental trials of the test and retest phases of the agent (panel a) and non-agent (panel b) conditions.

SI Figure 1.


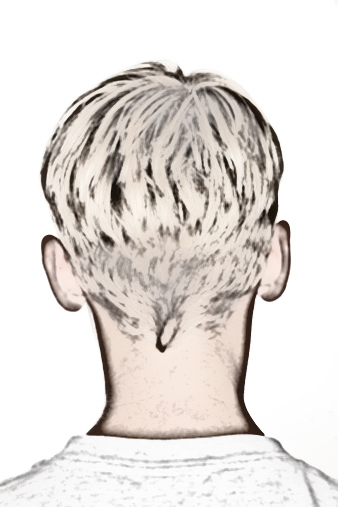

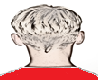
 xx A B

Note. Both child A and child B sit passively in their respective compartments whilst the rewards are delivered by Ghost A, controlled remotely by the experimenter. The black circles represent the lower value rewards (plain black stickers) and the white circle the higher value reward (colorful sticker).

SI Figure 2.

a Self-centered control


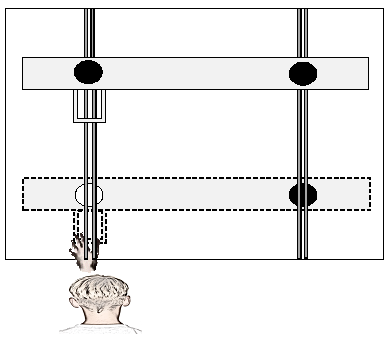


b Empty control


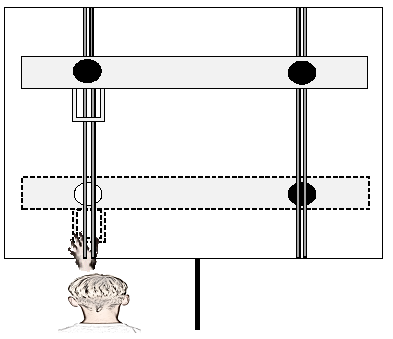


Note. In the self-centered control trials (panel a) the partition is removed allowing the child to access the rewards from both the donor and receiver compartments. In the empty control trials (panel b) the partition is in place and the child can only access the rewards available on the donor’s side of the apparatus. In each panel the black circles represent the lower value rewards (plain black stickers) and the white circle represents the higher value reward (colorful sticker).

SI Figure 3.


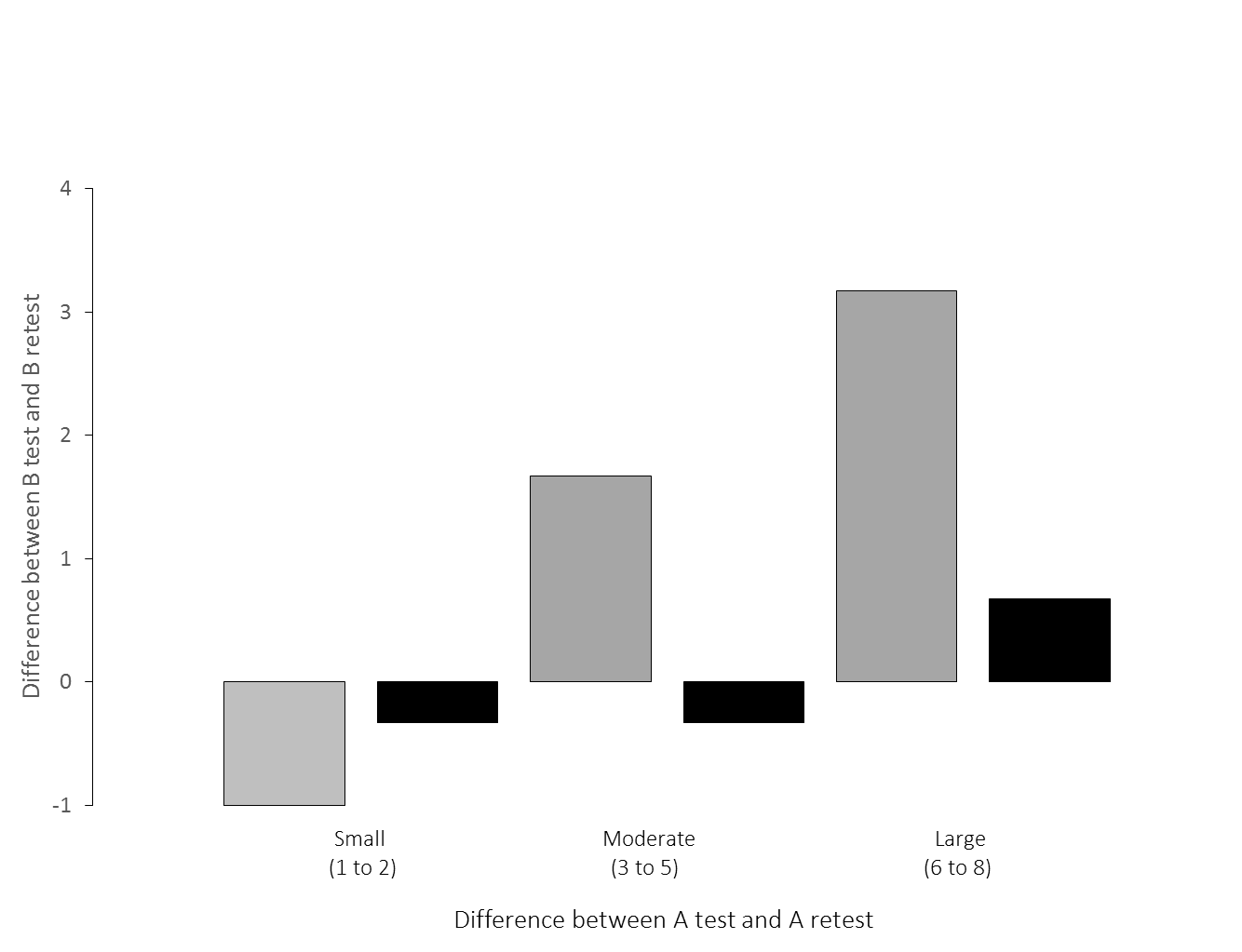


Note. Performance in the agent condition is shown by dark gray bars and performance in the non-agent condition by black bars.

SI Figure 4.


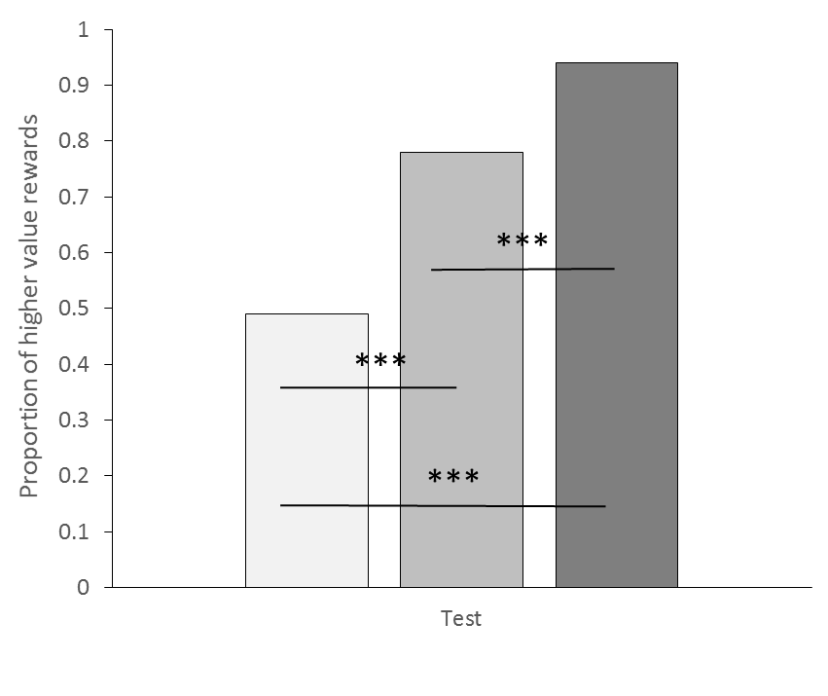

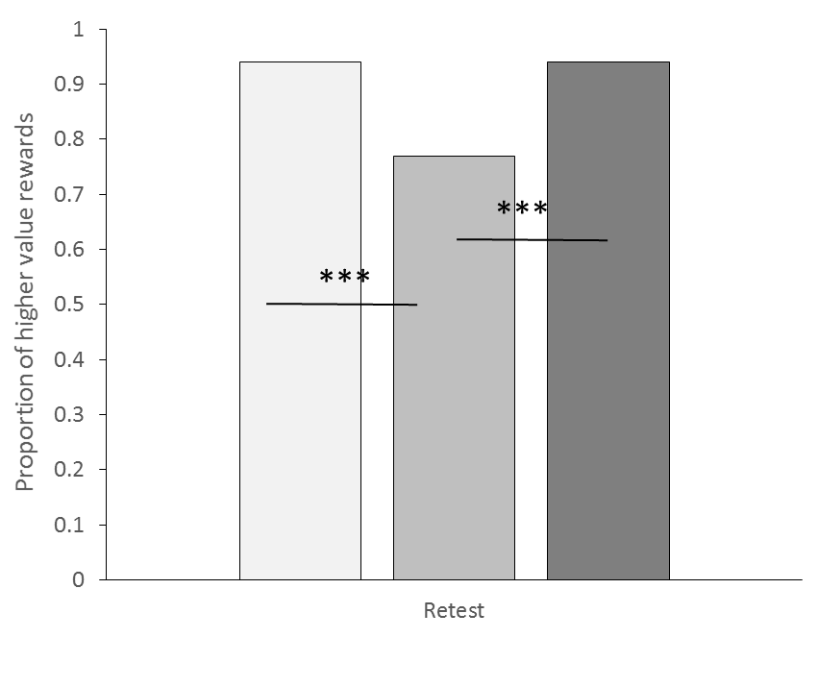


a Test Phase b Retest Phase

E EC SC E EC SC


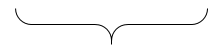

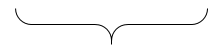


Controls Controls

Note. E = experimental trials, EC = empty control trials, SC = Self-centered control trials. *** p < .001

SI Figure 5.


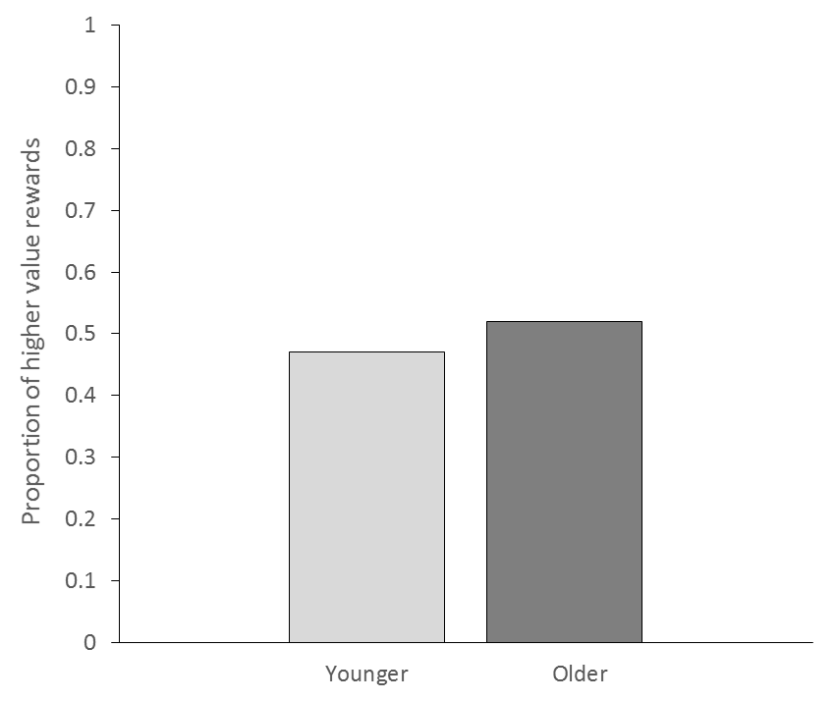


SI Figure 6.


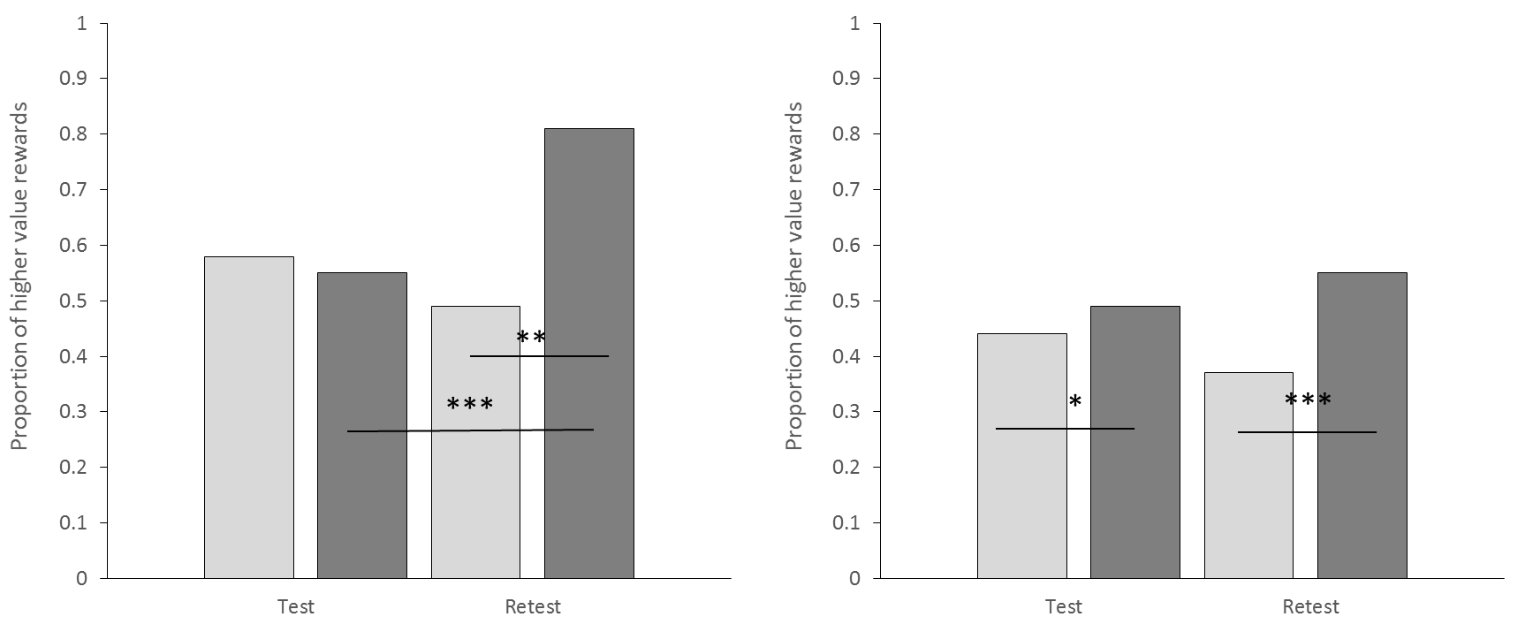


a Agent condition b Non agent Condition

Note. The performance of the younger children is shown with light gray bars and the performance of the older children by dark gray bars. *** p < .001, ** p < .01, * p < .05

**Supplementary Information: Tables**

SI Table 1. Coefficients of the model predicting B’s level of prosocial donating at test and retest in the non-agent condition [95% BCa bootstrap confidence intervals based on 1000 samples]

|  | b | 95% CI for Odds Ratio | | |
| --- | --- | --- | --- | --- |
|  |  | Lower | Odds | Upper |
| **Test Phase** |  |  |  |  |
| Included |  |  |  |  |
| Constant | -.90 |  |  |  |
|  | [-3.48, 1.43] |  |  |  |
| Ghost A test | .25 |  |  |  |
|  | [-.37, .82] | .40 | 1.28 | 2.26 |
|  |  |  |  |  |
| Child B’s age | .01  [-.02, .04] | .98 | 1.01 | 1.04 |
| **Retest Phase** |  |  |  |  |
| Included |  |  |  |  |
| Constant | -2.92 |  |  |  |
|  | [-5.63, -.73] |  |  |  |
| Ghost A test | .21 | .48 | 1.23 | 2.21 |
|  | [-.38, .82] |  |  |  |
| Child B test | .63* | 1.00 | 1.03 | 1.06 |
|  | [.00, 1.24] |  |  |  |
|  |  |  |  |  |
| Child B’s age | .03*  [.00, 1.24] | 1.05 | 1.88 | 3.37 |
|  |  |  |  |  |

Note. Test R² = .005 (Cox & Snell), .007 (Nagelkerke). Model χ²(2) = 1.04, p = .59

Note. Retest R² = .05 (Cox & Snell), .07 (Nagelkerke). Model χ²(3) = 9.55, p = .02

Note. * p < .05

SI Table 2. Coefficients of the model predicting A’s level of prosocial donating in the control and experimental trials at test and retest in the agent condition [95% BCa bootstrap confidence intervals based on 1000 samples]

|  | b | 95% CI for Odds Ratio | | |
| --- | --- | --- | --- | --- |
|  |  | Lower | Odds | Upper |
| **Test Phase** |  |  |  |  |
| Included |  |  |  |  |
| Constant | -.43 |  |  |  |
|  | [-.84, -.03] |  |  |  |
| Trial type | .60*** | 1.56 | 1.81 | 2.12 |
|  | [.43, .76] |  |  |  |
|  |  |  |  |  |
| **Retest Phase** |  |  |  |  |
|  |  |  |  |  |
| Included |  |  |  |  |
| Constant | 6.20 |  |  |  |
|  | [4.31, 9.07] |  |  |  |
| Trial type | -.60*** | .42 | .55 | .72 |
|  | [-1.0, -.33] |  |  |  |

Note. Test R² = .10 (Cox & Snell), .14 (Nagelkerke). Model χ²(1) = 59.42, p < .001

Note. Retest R² = .04 (Cox & Snell), .08 (Nagelkerke). Model χ²(1) = 24.16, p < .001

Note. *** p < .001

SI Table 3. Coefficients of the model predicting B’s level of prosocial donating in the control and experimental trials at test and retest in the agent condition [95% BCa bootstrap confidence intervals based on 1000 samples]

|  | b | 95% CI for Odds Ratio | | |
| --- | --- | --- | --- | --- |
|  |  | Lower | Odds | Upper |
| **Test Phase** |  |  |  |  |
| Included |  |  |  |  |
| Constant | -.74 |  |  |  |
|  | [-1.55, .04] |  |  |  |
| Trial type | .64*** | 1.48 | 1.89 | 2.42 |
|  | [.36, .93] |  |  |  |
|  |  |  |  |  |
| **Retest Phase** |  |  |  |  |
| Included |  |  |  |  |
| Constant | .01 |  |  |  |
|  | [-1.99, 2.07] |  |  |  |
| Trial type | .19 | .94 | 1.21 | 1.54 |
|  | [-.10, .47] |  |  |  |

Note. Test R² = .05 (Cox & Snell), .07 (Nagelkerke). Model χ²(1) = 27.44, p < .001

Note. Retest R² = .004 (Cox & Snell), .006 (Nagelkerke). Model χ²(1) = 2.25, p = .134

Note. *** p <.001

SI Table 4. Coefficients of the model predicting B’s level of prosocial donating in the control and experimental trials at test and retest in the non-agent condition [95% BCa bootstrap confidence intervals based on 1000 samples]

|  | b | 95% CI for Odds Ratio | | |
| --- | --- | --- | --- | --- |
|  |  | Lower | Odds | Upper |
| **Test Phase** |  |  |  |  |
| Included |  |  |  |  |
| Constant | .72 |  |  |  |
|  | [.47, 1.04] |  |  |  |
| Trial type | -1.14*** | 1.62 | 2.06 | 2.62 |
|  | [-2.0, -.40] |  |  |  |
|  |  |  |  |  |
| **Retest Phase** |  |  |  |  |
| Included |  |  |  |  |
| Constant | .82 |  |  |  |
|  | [.55, 1.15] |  |  |  |
| Trial type | -4.66*** | 1.77 | 2.26 | 2.89 |
|  | [-6.82, -2.78] |  |  |  |

Note. Test R² = .06 (Cox & Snell), .09 (Nagelkerke). Model χ²(1) = 37.37, p < .001

Note. Retest R² = .08 (Cox & Snell), .11 (Nagelkerke). Model χ²(1) = 46.61, p < .001

Note. *** p < .001

SI Table 5. Coefficients of the model predicting A’s level of prosocial donating at test in the agent condition [95% BCa bootstrap confidence intervals based on 1000 samples]

|  | b | 95% CI for Odds Ratio | | |
| --- | --- | --- | --- | --- |
|  |  | Lower | Odds | Upper |
| **Test Phase** |  |  |  |  |
| Included |  |  |  |  |
| Constant | 1.0 |  |  |  |
|  | [-1.41, 3.11] |  |  |  |
| Child A’s age | -.01 | .96 | .99 | 1.03 |
|  | [-.04, .03] |  |  |  |
| Friendship | .08 | .53 | 1.08 | 2.18 |
|  | [-.59, .79] |  |  |  |
| Dyad gender | -.26 | .57 | .78 | 1.05 |
|  | [-.58, .05] |  |  |  |

Note. R² = .02 (Cox & Snell), .03 (Nagelkerke). Model χ²(3) = 3.47, p = .33

SI Table 6. The proportion of higher value rewards allocated by child A and child B as a function of dyad relationship and dyad gender.

|  | Test | | Retest | |
| --- | --- | --- | --- | --- |
|  | Agent | Non-agent | Agent | Non-agent |
| **Child A** |  |  |  |  |
| *Relationship* |  |  |  |  |
| Friend | .47 | - | - | - |
| Acquaintance | .45 | - | - | - |
|  |  |  |  |  |
| *Dyad gender* |  |  |  |  |
| F-F | .53 | - | - | - |
| M-M | .56 | - | - | - |
| M-F | .40 | - | - | - |
| F-M | .44 | - | - | - |
|  |  |  |  |  |
| **Child B** |  |  |  |  |
| *Relationship* |  |  |  |  |
| Friend | .60 | .51 | .71¹ | .40 |
| Acquaintance | .48 | .44 | .52 | .57 |
|  |  |  |  |  |
| *Dyad gender* |  |  |  |  |
| F-F | .60 | .56 | .67 | .43 |
| M-M | .46 | .35 | .44² | .43 |
| M-F | .75 | .44 | .72 | .47 |
| F-M | .35 | .44 | .90³ | .50 |

Note. ¹Indicates significantly higher level of prosocial donation to friends at retest. ²Indicates significantly lower level of prosocial donation by M-M pairs as compared to other pairs at retest. ³Indicates significantly higher level of prosocial donation by F-F pairs as compared to other pairs at retest

SI Table 7. Coefficients of the model predicting B’s level of prosocial donating at test and retest in the agent condition [95% BCa bootstrap confidence intervals based on 1000 samples]

|  | b | 95% CI for Odds Ratio | | |
| --- | --- | --- | --- | --- |
|  |  | Lower | Odds | Upper |
| **Test Phase** |  |  |  |  |
| Included |  |  |  |  |
| Constant | 1.55 |  |  |  |
|  | [-.74, 4.23] |  |  |  |
| Child B’s age | -.02 | .95 | .98 | 1.01 |
|  | [-.05, .01] |  |  |  |
| Friendship | .48 | .83 | 1.62 | 3.17 |
|  | [-.23, 1.12] |  |  |  |
| Dyad gender | -.11 | .66 | .89 | 1.21 |
|  | [-.43, .21] |  |  |  |
|  |  |  |  |  |
| *** p < .001 |  |  |  |  |
| Included |  |  |  |  |
| Constant | -6.75 |  |  |  |
|  | [-11.17, -4.07] |  |  |  |
| Child B’s age | .06** | 1.02 | 1.06 | 1.11 |
|  | [.03, .12] |  |  |  |
| Friendship | 1.09* | 1.26 | 3.00 | 7.08 |
|  | [.26, 2.12] |  |  |  |
| Dyad gender | 1.0*** | 1.74 | 2.71 | 4.21 |
|  | [.59, 1.73] |  |  |  |

Note. Test R² = .02 (Cox & Snell), .03 (Nagelkerke). Model χ²(3) = 4.08, p = .25

Note. Retest R² = .23 (Cox & Snell), .32 (Nagelkerke). Model χ²(3) = 44.08, p < .001

Note. * p < .05, **p < .01 ***p < .001

SI Table 8. Coefficients of the model predicting B’s level of prosocial donating at test and retest in the non-agent condition [95% BCa bootstrap confidence intervals based on 1000 samples]

|  | b | 95% CI for Odds Ratio | | |
| --- | --- | --- | --- | --- |
|  |  | Lower | Odds | Upper |
| **Test Phase** |  |  |  |  |
| Included |  |  |  |  |
| Constant | -3.16 |  |  |  |
|  | [-6.21, -.50] |  |  |  |
| Child B’s age | .04* | 1.01 | 1.04 | 1.08 |
|  | [.01, .08] |  |  |  |
| Friendship | .34 | .72 | 1.40 | 2.74 |
|  | [-.39, 1.04] |  |  |  |
| Dyad gender | -.09 | .67 | .91 | 1.24 |
|  | [-.44, .22] |  |  |  |
|  |  |  |  |  |
| **Retest Phase** |  |  |  |  |
| Included |  |  |  |  |
| Constant | -4.67 |  |  |  |
|  | [-8.11, -1.74] |  |  |  |
| Child B’s age | .07*** | 1.03 | 1.07 | 1.12 |
|  | [.04, .12] |  |  |  |
| Friendship | -.65 |  |  |  |
|  | [-1.48, .03] | .26 | .52 | 1.05 |
| Dyad gender | -.16 |  |  |  |
|  | [-.60, .19] | .61 | .85 | 1.19 |

Note. Test R² = .04 (Cox & Snell), .05 (Nagelkerke). Model χ²(3) =6.37, p = .10

Note. Retest R² = .12 (Cox & Snell), .16 (Nagelkerke). Model χ²(3) = 20.12, p < .001
